# Management of neurogenic bladder in myelodysplasia patients: experience at our center

**DOI:** 10.1186/1743-8454-7-S1-S8

**Published:** 2010-12-15

**Authors:** Sangram Singh, Milind Joshi, Vigya Chourishi

**Affiliations:** 1 Department of Pediatric Surgery, Sri Aurobindo Institute of Medical Sciences, Indore. M.P, India

## Background

Myelodysplasia is the term used for lesions include Spina bifida occulta, lipomeningocoele, meningocoele and meningomyelocele. meningomyelocoele is by far the most common disease of this group. These patients are commonly having neurogenic bladder. The treatment of neurogenic bladder is not so easy.[1] we are presenting our experience of 68 cases with neurogenic bladders. The aim of our study is to achieve continence and preserve upper urinary tract from further damage. There are a battery of investigations to assess these patients for eg. renal biochemistry, ultrasonography of the urinary tract, Micturating cysto-urethrogram,isotope renal scans, Clinical and bedside assessment, and Urodynamics.Tratment options available for these patients are drug therapy in the form of prophylactic antibiotics and anticholinergic drugs. Clean intermittent catheterization advocated by Lapides plays major role in the treatment of these patients [2]. Operative procedure perform in these patients are bladder neck reconstructions like, Young Dees Leadbetter and rectus sling.[3] High pressure bladder can be converted to low pressure bladder by using bladder augmentation procedures. Bladder augmentation procedures are increasing bladder capacity also thus improving dry intervals. Continent urinary diversion can be done by using appendix [4] and segment of ileum [5]. Once these patients achieve social or urinary continence follow up is very important.

## Material and methods

We at our center in India have evaluated 68 patients of spinal dysraphism following standard protocols. Out of these 68 patients 45 were having urinary incontinence. 34 patients were advised CIC and oxybutynin from second months onward. Nine patients underwent continent urinary diversion using Mitrofanoff's(8 patients)and Monti's 1 principle. Two patients underwent rectus sling bladder neck suspension and now on CIC.

Patients who underwent continent urinary diversion are doing well and maintaining good dry intervals. One patient who underwent Mitrofanoff's procedure developed post-operative necrosis of the ileal patch along with appendix due to severe lordosis. 6 patients are totally dry, and one patient developed post operative wound infection. The upper tract of these patients is okay, but long term follow up will be required to draw further outcome.

## Conclusions

Proper assessment and appropriate medical and surgical intervention is must to achieve continence in neurogenic bladder patients.
